# Patient-Reported Outcomes in Systemic Lupus Erythematosus. Can Lupus Patients Take the Driver’s Seat in Their Disease Monitoring?

**DOI:** 10.3390/jcm11020340

**Published:** 2022-01-11

**Authors:** Ioannis Parodis, Paul Studenic

**Affiliations:** 1 Division of Rheumatology, Department of Medicine Solna, Karolinska Institutet and Karolinska University Hospital, 171 76 Stockholm, Sweden; paul.studenic@ki.se; 2 Department of Rheumatology, Faculty of Medicine and Health, Örebro University, 701 82 Örebro, Sweden; 3 Division of Rheumatology, Department of Internal Medicine 3, Medical University of Vienna, 1090 Vienna, Austria

**Keywords:** systemic lupus erythematosus, patient-reported outcomes, patient perspective, health-related quality of life, shared decision, person-centred care

## Abstract

Systemic lupus erythematosus (SLE) is a chronic autoimmune disorder that has detrimental effects on patient’s health-related quality of life (HRQoL). Owing to its immense heterogeneity of symptoms and its complexity regarding comorbidity burden, management of SLE necessitates interdisciplinary care, with the goal being the best possible HRQoL and long-term outcomes. Current definitions of remission, low disease activity, and response to treatment do not incorporate self-reported patient evaluation, while it has been argued that the physician’s global assessment should capture the patient’s perspective. However, even the judgment of a very well-trained physician might not replace a patient-reported outcome measure (PROM), not only owing to the multidimensionality of self-perceived health experience but also since this notion would constitute a direct contradiction to the definition of PROMs. The proper use of PROMs is not only an important conceptual issue but also an opportunity to build bridges in the partnership between patients and physicians. These points of consideration adhere to the overall framework that there will seldom be one single best marker that helps interpret the activity, severity, and impact of SLE at the same time. For optimal outcomes, we not only stress the importance of the use of PROMs but also emphasize the urgency of adoption of the conception of forming alliances with patients and facilitating patient participation in surveillance and management processes. Nevertheless, this should not be misinterpreted as a transfer of responsibility from healthcare professionals to patients but rather a step towards shared decision-making.

## 1. Introduction

Systemic lupus erythematosus (SLE) is a chronic autoimmune disorder that has detrimental effects on a patient’s health-related quality of life (HRQoL) [1]. It is widely known that SLE is a rheumatic condition that is challenging to diagnose and treat, mainly owing to its immense heterogeneity of clinical symptoms and complexity with regard to comorbidity burden, often necessitating interdisciplinary care, with the goal being the best possible quality of life and long-term outcomes [2,3]. With the premise that preventing is better than restoring, early diagnosis and treatment initiation is imperative [4], a need of particular urgency given that up to 10% of SLE patients develop life-threatening conditions or complications, such as end-stage kidney disease [5,6]. Patient-reported outcome measures (PROMs) receive increasing attention within the lupus research community, especially PROMs addressing HRQoL [7]. Even though this signifies a shift of the current paradigm towards increasing patient participation in their care, more distance has to be bridged before PROMs are an integral part of the evaluation in clinical practice.

## 2. PROMs in the Treat-to-Target Context

Remission of systemic symptoms and organ manifestations was identified by the treat-to-target for SLE initiative (T2T/SLE) as one of the most important targets in the management of patients with SLE [8]. Several definitions of remission were used in clinical trials and observational studies of SLE [9]. The Definitions Of Remission In SLE (DORIS) is an international task force consisting of expert rheumatologists, nephrologists, dermatologists, clinical immunologists, and patient representatives, who jointly proposed a set of remission definitions in response to the T2T/SLE research agenda [10]. Later, the group decided on one prevailing remission definition that incorporated a physician-reported global assessment (PhGA), the SLE Disease Activity Index 2000 (SLEDAI-2K) [11] after suppression of the serological descriptors (anti-double-stranded (ds)DNA and complement levels), the current daily glucocorticoid dose, and restrictions regarding medication allowance. Practically, however, some patients’ individual situations make it hard for them to achieve this stringent target. In such cases, one should aim for low disease activity (LDA). Again, several definitions of LDA have been proposed in the literature, with the Lupus Low Disease Activity State (LLDAS) [12] being the most commonly used.

**Figure 1:**
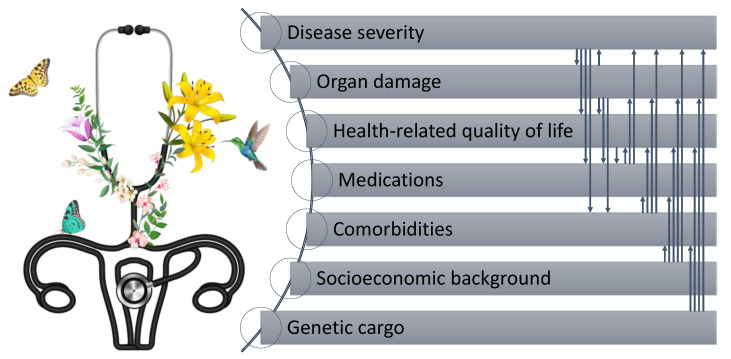
Illustration of the multilateral impact across disease facets and health-related quality of life in people living with systemic lupus erythematosus.

In addition, several recent clinical trials of SLE have used composite indices to define response to treatment, e.g., the SLEDAI Responder Index (SRI) [13] or the British Isles Lupus Assessment Group (BILAG)-based Combined Lupus Assessment (BICLA) [14]. Notably, none of the proposed definitions of remission, LDA, or response to treatment incorporate self-reported patient evaluation, which may constitute a pitfall of the definitions. Indeed, the DORIS task force discussed the issue of the non-inclusion of a PROM and partwise argued that the PhGA should incorporate the patient’s perspective by paying careful attention to the patient’s symptoms and experience [15]. However, even the judgment of a very well-trained physician might not replace a PROM, not only owing to the multidimensionality of patient-perceived health experience (Figure 1), but also since this notion would constitute a direct contradiction to the definition of a PROM; patient-reported outcomes are directly recorded by patients, without interpretation by their clinicians, and are additional markers in the assessment of treatment impact [16,17]. In the definition of remission in rheumatoid arthritis (RA), the patient’s global assessment (PtGA) was chosen as one of the four Boolean criteria because of its ability to show a large sensitivity to change and its discriminatory validity between active drug and placebo in clinical trials [15]. However, it is not easy to address the question of whether PtGA is an ideal PROM to complement the current definition of remission. In fact, a debate has been ongoing for a decade with regard to the appropriateness of PtGA to be included in the remission definition in RA, at least with the currently used cut-offs, since substantial proportions of patients score their disease activity higher than their rheumatologists [18,19,20]. Due to the lack of evidence-based alternatives to PtGA, a summary report of an Outcome Measures in Rheumatology (OMERACT) interest group recently stated that currently, no better tools for representing the patients’ perspective are available, thus the definition should be kept as is [21]. It is, however, worth noting that the differences in heterogeneity and complexity between SLE and RA are also reflected in discrepant longitudinal patterns of self-reported health experience [22] and direct comparisons or extrapolations of psychometric properties of PROMs between the two diseases may be misleading.

The issue of imperfect agreement in the perception of disease severity between patients and physicians was also addressed in the field of SLE. While LDA is coupled with an overall favorable HRQoL experience, at least when assessed with the LLDAS [23], results from other investigations are conflicting. One study used a Systemic Lupus Activity Questionnaire (SLAQ) score < 6 as the cut-off for LDA as perceived by the patients and found that only one-quarter of patients who were classified as being in LLDAS fulfilled the definition of SLAQ score < 6 [24]. In the same study, Medical Outcomes Survey Short Form 36 (SF-36) component summary scores and Functional Assessment of Chronic Illness Therapy Fatigue scale (FACIT-F) scores were higher among patients in LLDAS who reported SLAQ scores < 6 than among patients in LLDAS who reported SLAQ scores ≥ 6 [24]. This not only underscores that the physician’s perspective does not entirely represent the patient’s perception of HRQoL, but also highlights that LLDAS alone may not be a sufficient target in the management of people with SLE. The inclusion of HRQoL measures in treatment evaluation processes was supported by the T2T statements and received additional weight through findings indicating that poor HRQoL is associated with increased mortality [8,25]. To this end, it is important to clarify that this discussion is not intended to devalue the PhGA or the rheumatologists’ ability to perform adequate clinical judgment; in fact, PhGA scores have shown good correlates with mental health, overall disease activity, and flares [26].

## 3. The Matter of Not Only Optimal Choice but Also Optimal Use of PROMs

The OMERACT IV consensus conference [27] propounded disease activity, HRQoL, medication side-effects, and organ damage as the four core outcomes for SLE clinical trials in that priority order. In light of accumulating evidence of the discordance in perceptions of disease activity between physicians and patients with SLE [28], PROMs are increasingly used in SLE clinical trials [7]. The SF-36 [29] and FACIT-F [30] were reviewed for their psychometric properties with regard to the extent to which they comply with the US Food and Drug Administration (FDA) guidance [31] and were suggested as secondary endpoints to support the labeling of novel therapies for SLE [32]. Changes in scores in various SF-36 domains and FACIT-F have shown an ability to discriminate between verum drug (belimumab) and placebo in the BLISS-52 and BLISS-76 clinical trials [33]. In the same analysis, changes in EQ-5D utility index scores [34] did not exhibit discriminative ability. However, in light of satisfactory psychometric properties of EQ-5D for SLE patients, particularly in terms of validity and reliability [35], a recent study investigated the discriminative ability and known-group validity of EQ-5D full health state (FHS), i.e., a utility index score of 1, and found a remarkably robust ability of EQ-5D FHS to discriminate between drug and placebo, and between responders and non-responders in the BLISS-52 and BLISS-76 clinical trials [36]. This not only illustrates the need for determining optimal PROMs in SLE but also supports the notion that optimal use of the currently available ones may be even more important. For example, the differential ability of PROMs to capture changes in the different SLE disease patterns, i.e., persistently quiescent, persistently relapsing-remitting, and persistently active disease [37], comprises one of the many questions framing the future research agenda.

## 4. Can Lupus Patients Take the Driver’s Seat in Their Disease Monitoring?

Several questions regarding the validity of PROMs for people with SLE were clarified over the past decades [38,39]. We outlined their value in the interpretation of trial data for therapies that are potentially beneficial in the management of SLE. It is apparent that treatment cannot be narrowed down to the pharmacological component only, when the goal is remission on the one hand and a state of good or acceptable HRQoL for people living with SLE on the other. Knowing that SLE influences multiple domains of life, the use of generic and disease-specific measures in structured evaluation processes is justified. In this regard, it is worth noting that mainly generic PROMs have been used in randomized clinical trials of SLE, particularly the SF-36, while instruments with the ability to discriminate across clinically distinct groups are found to be more responsive to change, lending support for broader use of disease-specific PROMs [40]. The SLE-specific SLAQ is designed to capture self-reported symptoms that are usually evaluated by the rheumatologist [41] and is based on the Systemic Lupus Activity Measure [42]. While it shows adequate reliability and correlates with SF-36, it is not always congruous with traditional clinical parameters [43]. The Lupus Patient-Reported Outcome tool (LupusPRO) entails several domains of HRQoL and additionally prompts patients to reflect on support, medications, satisfaction, and cognition [44]. The LupusPRO correlates with the SF-36 and shows responsiveness in relation to the physician-reported activity index BILAG [45]. However, a systematic comparison across available PROMs in relation to generic domains of HRQoL and SLE-specific symptoms to determine the best correlates with traditional physician-reported disease features has yet to be conducted.

Assessments and interventions need to be tailored to the individual patient and decided upon together with the patient. In fact, shared decision-making constitutes a primary overarching principle of the T2T/SLE task force recommendations [8]. For some people with SLE, the impact of the disease acts as a negative spiral, in particular with regard to mental health, whereas high pain and global disease burden are coupled with negative future outcomes [46]. Physical or functional domains of HRQoL may still be contracted in considerable proportions of patients irrespective of their overall response to treatment, which has been shown to be more prominent in patients with established organ damage, and furthermore, dependent on ethnicity [47]. PROMs are scored worse in people with lower health literacy, which is not necessarily related to lower income or education, albeit resources of people with lower socioeconomic backgrounds are under stronger constraints [48,49]. It is also important to bear in mind that comorbidities may have an impact on how patients score their HRQoL, e.g., depressive or other disorders causing chronic mental distress may impact on pain, fatigue, or PtGA scores [46,50]. Thus, persistently high scores or persistent discordance between patient’s and physician’s assessments should intrinsically prompt further investigation for potential comorbid conditions as underlying causes and, if needed, the commencement of suitable adjunct therapy. Nevertheless, the considerable variability of PhGA scoring across assessors highlights the need to adopt optimal tools and determine the optimal timing for the assessment, as well as integrate multiple items, including the patient perspective, with the ultimate goal being a holistic apprehension of the disease status [51,52].

The proper use of PROMs is not only an important conceptual issue but also an opportunity to build bridges in the partnership between patients and physicians. These points of consideration adhere to the overall framework that there will seldom be one single best marker that helps us to interpret the activity, severity, and impact of SLE at the same time. By contrast, in clinical practice, there is a battery of tests and assessment instruments that the healthcare team may choose from, based on what is relevant to the respective patient and the respective condition. However, harmonization and integration of different tests in surveillance and overall patient management should be supported by data and strive for optimization, taking environmental, personal, and disease-specific conditions into account.

## 5. Conclusions

As a concluding remark, for optimal outcomes, we not only stress the importance of the use of PROMs but also emphasize the urgency to adopt the concept of forming alliances with each individual patient and facilitating active patient participation in surveillance and management processes as an integral part within the clinical consultations, and continuously during the disease course. The positive impact of this mindset on patients’ lives has been explored more in-depth for people with inflammatory arthritis [53,54]; being a considerably more complex condition, it would be counterintuitive to anticipate holistic pertinence in the management of SLE without active patient involvement in all steps. Nevertheless, this should not be misinterpreted as a transfer of responsibility from healthcare professionals to patients, but should rather be considered a step towards shared decision-making, which in fact should impose responsibility to healthcare to ensure adequate patient education and confidence in patients in understanding the need and the options. Thus, while time becomes more mature for patients with SLE to take the driver’s seat in their disease monitoring and management, healthcare professionals should not release themselves from responsibility but retain the seat of the navigator and inspirer.
